# A Unique Case of Acute Cerebral Venous Sinus Thrombosis Secondary to Primary Varicella Zoster Virus Infection

**DOI:** 10.7759/cureus.1693

**Published:** 2017-09-16

**Authors:** Syed F Imam, Omair ul haq Lodhi, Zainab Fatima, Saneeya Nasim, Waseem T Malik, Muhammad Sabih Saleem

**Affiliations:** 1 Department of Internal Medicine, Shifa International Hospital, Islamabad, Pakistan; 2 Shifa College of Medicine, Shifa International Hospital, Islamabad, Pakistan; 3 Medicine, Shifa International Hospital, Islamabad, Pakistan; 4 Department of Neurology, Shifa International Hospital, Islamabad, Pakistan

**Keywords:** varicella, cvt, vzv, cerebral venous thrombosis, venous thrombosis, chickenpox, cvst

## Abstract

Primary varicella zoster virus (VZV) infection, predominantly in the pediatric population, presents with pyrexia and a classic pruritic vesicular rash. In adults, although less common, it is more severe and linked to more complications. Neurological complications, which account for less than 1% of all VZV complications, include meningitis, encephalitis, arterial vasculopathy, and venous thrombosis. We present a case of a 39-year-old male who developed extensive cerebral venous sinus thrombosis following primary VZV infection. Venous thrombosis in VZV has been suggested to be caused by autoantibodies against protein S, pre-existing hypercoagulability, or endothelial damage. The patient was acutely managed using intravenous acyclovir and heparin. Long-term anticoagulation therapy with warfarin was continued after discharge. We concluded that clinicians should be aware of the rare complications of this common pathology so that a timely diagnosis can be made, followed by prompt management. Further studies need to be done to better understand acute cerebral venous sinus thrombosis secondary to VZV.

## Introduction

Primary varicella zoster virus (VZV) classically presents as a febrile illness in children with an exanthematous vesicular rash. The epidemiology of VZV is influenced by climate. In temperate climates, the incidence of VZV is 13-16 cases per 1,000 people per year. Greater than 90% of those are infected before adolescence. In tropical climates, VZV tends to occur later in life and adults are more prone to be affected than children [1]. Adults, however, frequently present with reactivation of dormant VZV virus instead of a primary infection. Primary VZV infection in adults tends to be more severe and is linked to greater complications. Neurological complications of VZV account for less than 1% of the affected population, causing meningitis, encephalitis, cerebellar ataxia, ventriculitis, ischemic or hemorrhagic stroke due to arterial vasculopathy, and rarely, venous thrombosis [2-3].

Very rarely, cerebral venous thrombosis secondary to primary VZV infection can occur due to a prothrombotic state [3]. We report the case of a 39-year-old man who developed extensive acute cerebral venous sinus thrombosis, a very rare complication of primary VZV infection.

## Case presentation

A 39-year-old male with a known case of chickenpox (primary VZV infection) of seven days duration presented at the emergency department (ED) with complaints of a headache and vomiting. The dull aching headache started five days prior in the occipital region, radiating to the neck, and was moderate to severe in intensity with no aggravating or relieving factors. Vomiting began two days prior, with six episodes per day, and no relief with medicines. The patient also had associated anorexia, lethargy, dizziness, and intermittent fever, varying between 99° F - 102° F for five days. No photophobia, shortness of breath, palpitations, or visual disturbances were noted. Prior medical, surgical, and family history were insignificant. Chickenpox, which started a week prior, was diagnosed on the basis of history and the clinical exam.

On initial examination, the patient was dehydrated and in discomfort. Scattered exanthematous vesicular lesions were observed all over his body, particularly on the face and trunk. His temperature was 100° F with a pulse of 70 beats per minute, blood pressure of 140/90 mmHg, respiratory rate of 18 breaths per minute, and a SpO2 of 97%. The patient's Glasgow Coma Score (GCS) was 15/15 and positive for slight neck stiffness; however, no weakness or neurological deficits were observed. The patient's strength was 5/5 in all four limbs with a complete range of motion, normal deep tendon reflexes, and a negative Babinski sign. 

**Figure 1:**
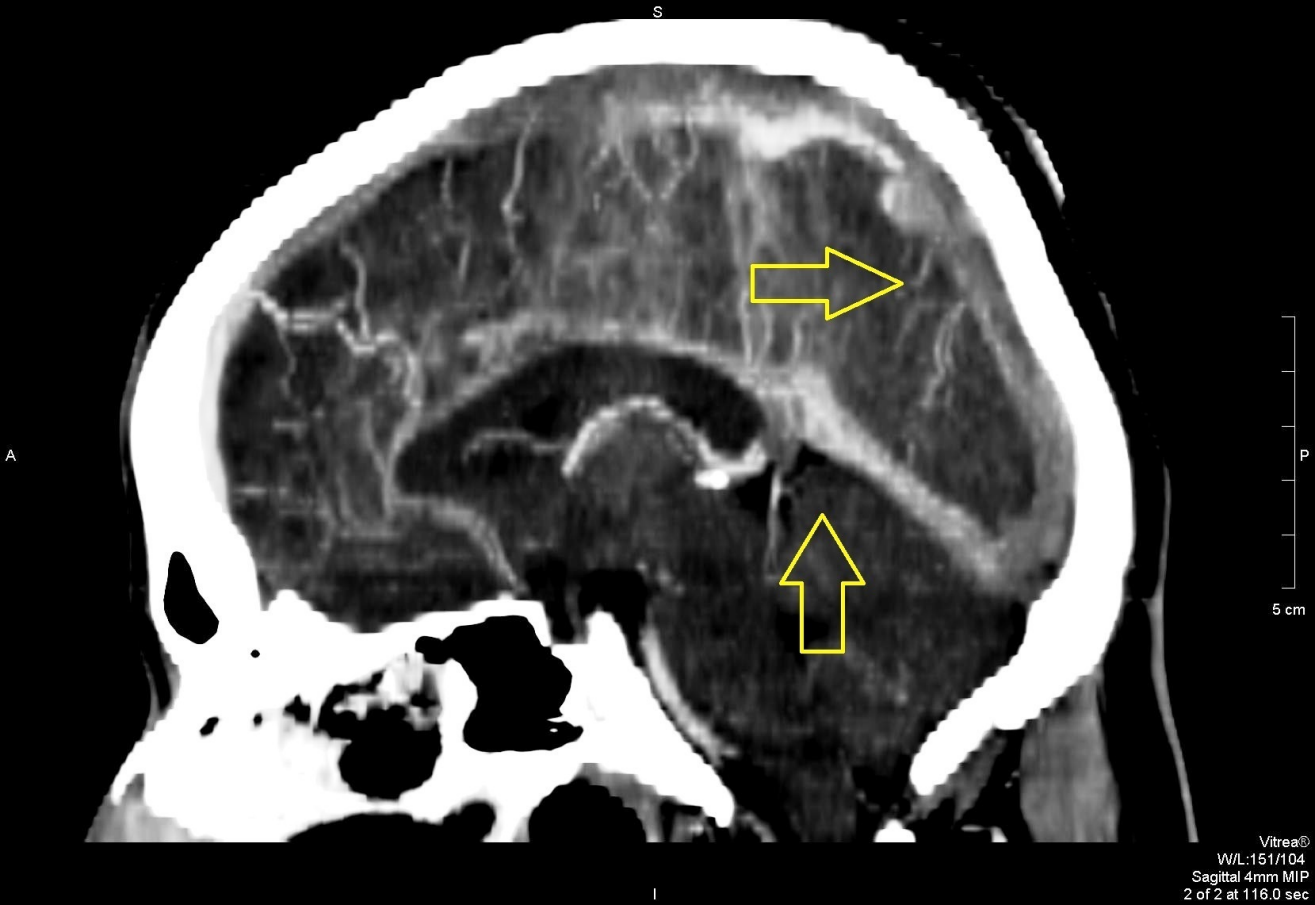
Sagittal view on contrast CT scan showing extensive sinus blockage CT: computed tomography

**Figure 2:**
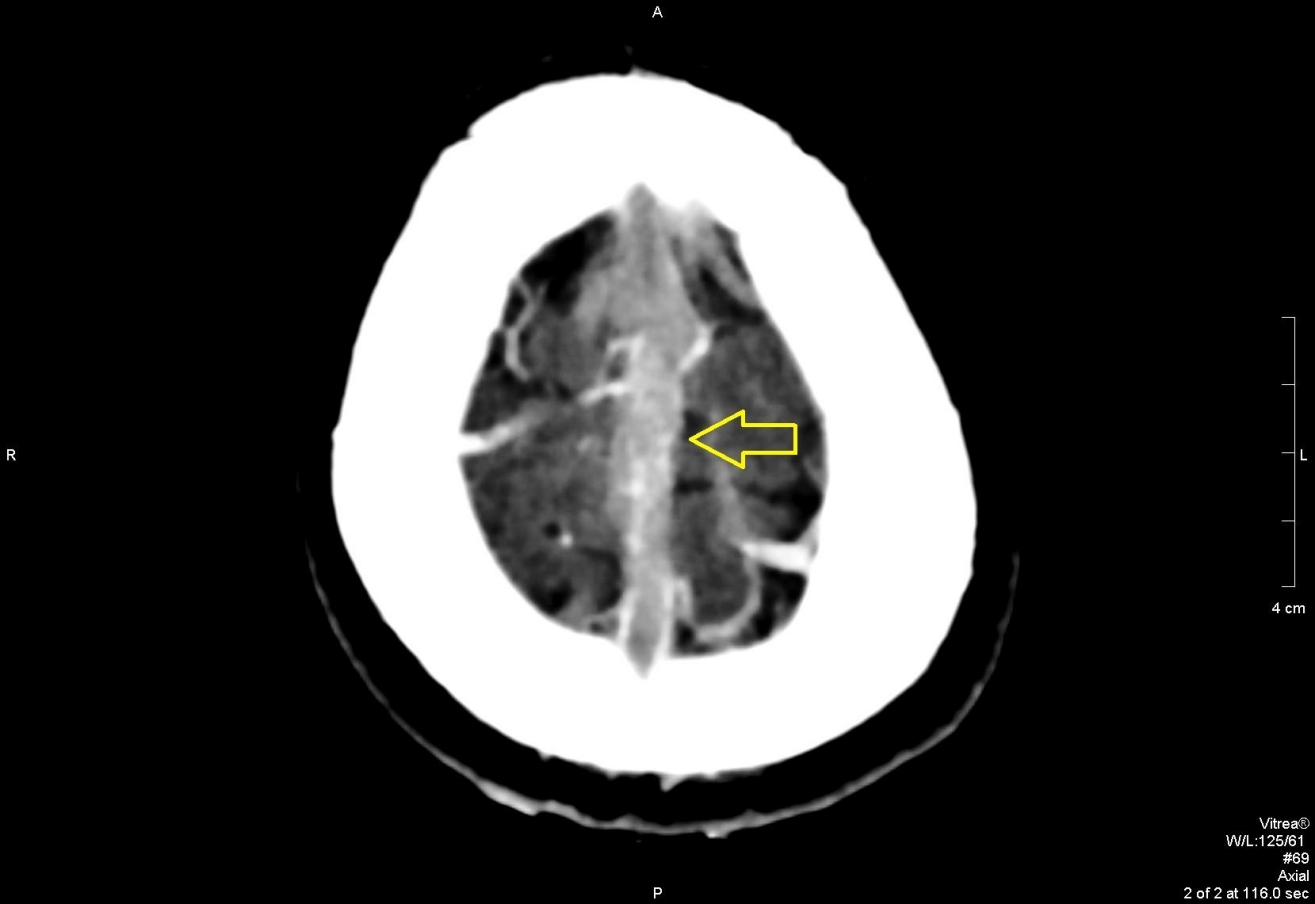
Axial view on contrast CT scan showing superior sagittal sinus blockage CT: computed tomography

**Figure 3:**
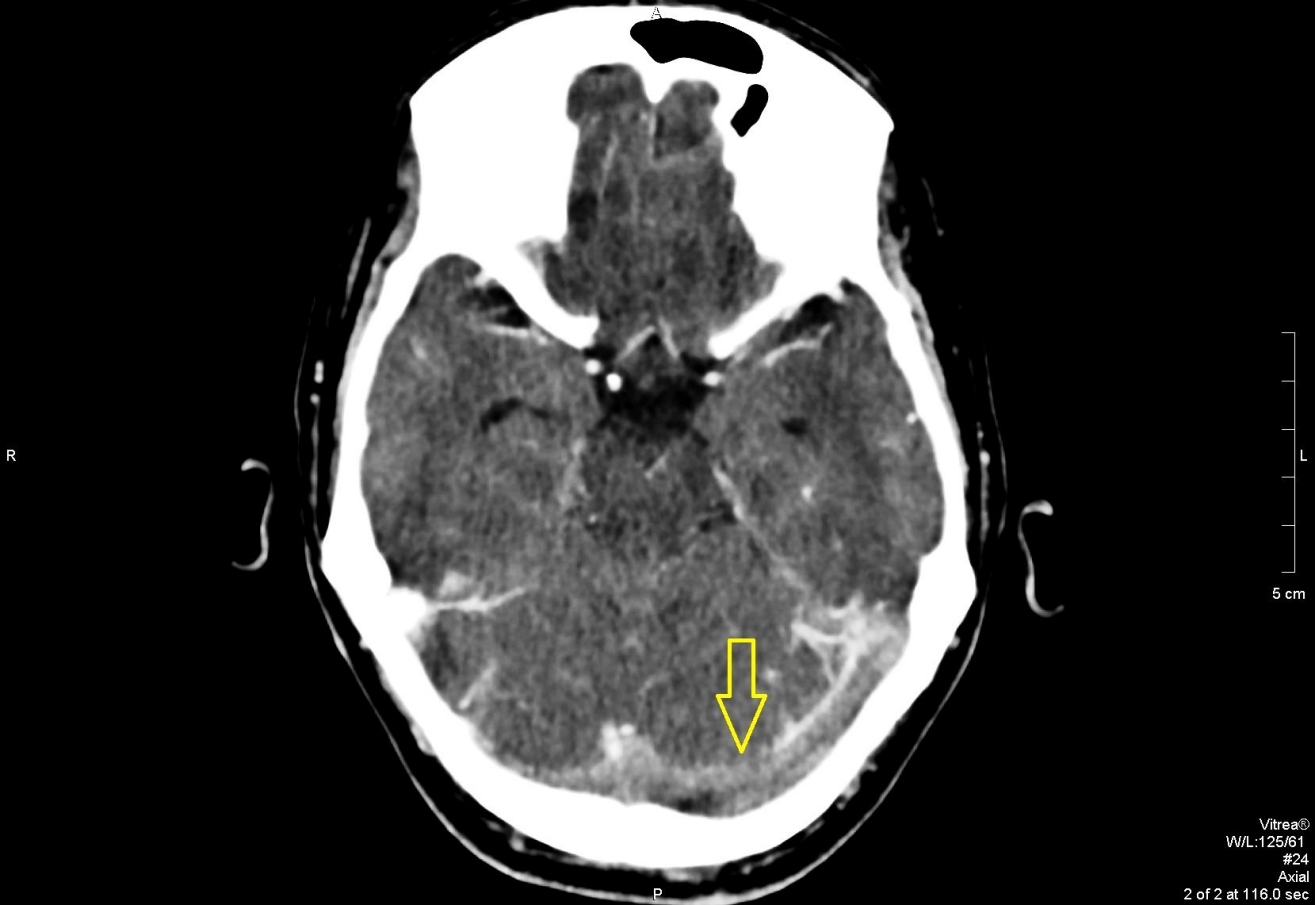
Axial view on contrast CT scan showing transverse sinus blockage CT: computed tomography

**Figure 4:**
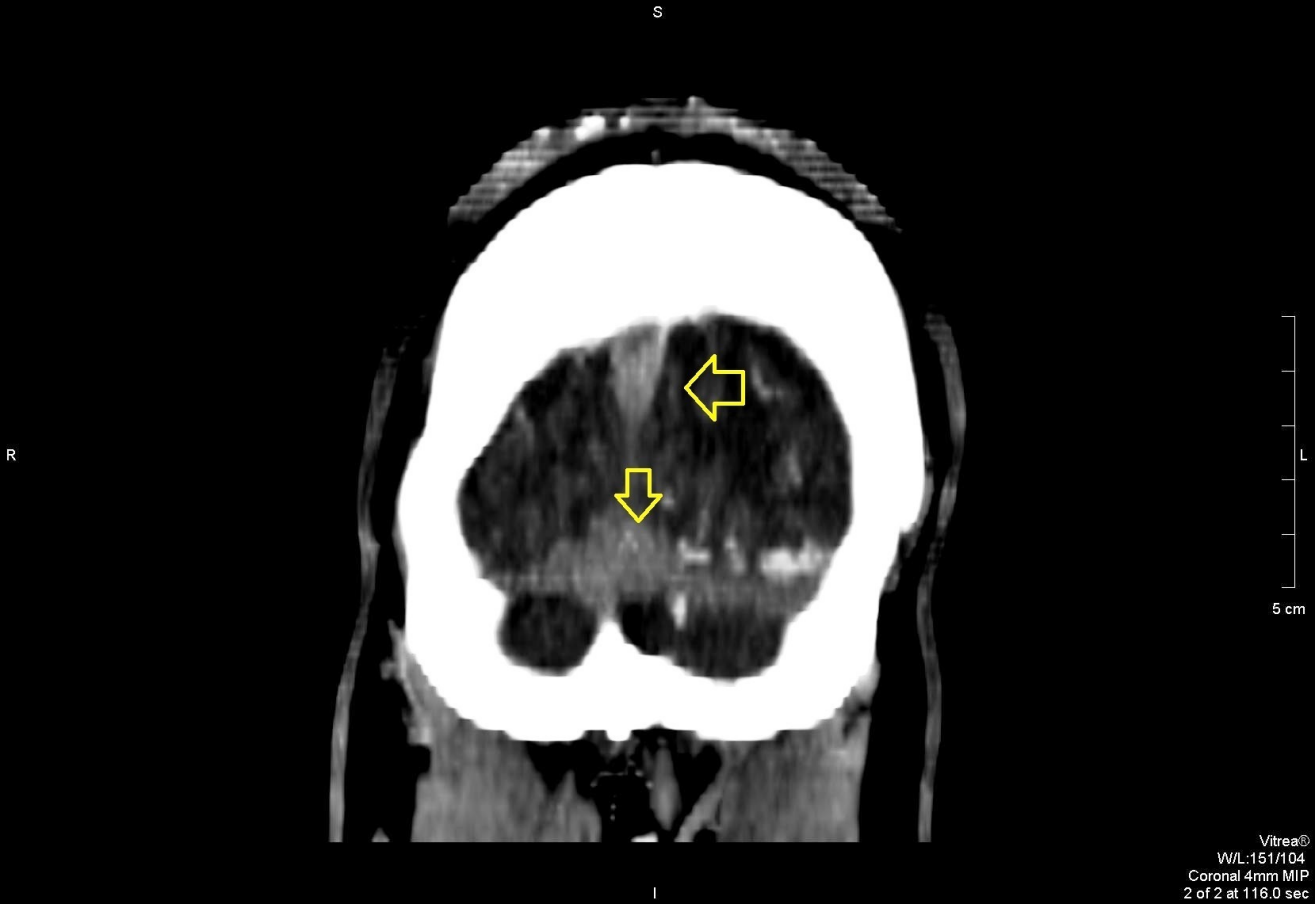
Coronal view on contrast CT scan showing superior sagittal sinus blockage and extensive thrombosis at the confluences of the sinuses CT: computed tomography

In the ED, further workup was done, which included serologic and radiologic investigations. A computed tomography (CT) scan revealed extensive cerebral venous sinus thrombosis, as shown in Figures 1-4. Intravenous acyclovir and anticoagulation therapy with heparin were started. The patient was urgently transferred to the intensive care unit where he was observed for the next few days

By day three, the patient started improving and there were no more active complaints of a headache, vomiting, or fever. The patient was also started on an adjusted dose of warfarin for long-term anticoagulation until a therapeutic international normalized ratio (INR) was attained. Intravenous acyclovir was discontinued after the course was completed, and heparin was bridged to warfarin for long-term anticoagulation. After a week of no active complaints, the patient was discharged in a stable condition with long-term anticoagulation therapy and a follow-up plan of care. The patient was also advised to return to the ED in case of any alarming symptoms in the future.

## Discussion

Primary VZV infection, also known as chickenpox, is a self-limiting febrile illness causing an exanthematous vesicular rash most commonly seen in children. Adults usually have reactivation of VZV as compared to primary varicella infection. Complications associated with VZV include encephalitis, cerebellar ataxia, transverse myelitis, ventriculitis, meningoencephalitis, and aseptic meningitis [2]. It can also be associated with ischemic stroke, carotid dissection, aneurysm, and subarachnoid or cerebral hemorrhages as a consequence of arterial vasculopathy. Rarely, VZV can also cause purpura fulminans and venous thrombosis due to a hypercoagulable state [4]. Our patient developed extensive cerebral venous sinus thrombosis (CVST), a very rare complication of primary VZV infection.

The basis of venous thrombosis can be linked to Virchow's triad, which includes injury to the vessel walls, a hypercoagulable state, and stasis [4]. The mechanism of venous thrombosis formation in post-primary VZV infection is hypothesized to occur due to either direct endothelial damage, inflammation of the vessel, induced autoantibodies against protein S, or a preexisting hypercoagulable state. In 2016, Paul, et al. stated that one out of three cases with post-varicella CVST had protein S deficiency, a preexisting hypercoagulable state [5]. In 2012, Siddiqi, et al. reported two cases of post-VZV CVST because of preexisting protein S and C deficiency [4]. According to a cross-sectional study done by Josephson, et al., 43 out of 95 children had antiphospholipid antibodies, while some had decreased protein S levels post-VZV infection. They called it varicella autoantibody syndrome [6].

CVST can present with isolated intracranial hypertension causing headache, vomiting, and papilledema. It may also present with convulsions, altered mental status, cranial nerve palsies, or focal deficits [7]. Our patient presented to the ED with only a headache and vomiting in addition to the ongoing fever and classical vesicular rash of primary VZV infection.  

Vasculopathies proceeding after VZV infection are often diagnosed using a cerebrospinal fluid polymerase chain reaction for anti-VZV immunoglobulin G antibodies and VZV deoxyribonucleic acid (DNA). Unless both are negative, vasculopathy cannot be ruled out [1]. If CVST is suspected, CT and magnetic resonance imaging (MRI) can reveal venous thrombosis or lack of flow in the cerebral veins, confirming the diagnosis. Additionally, protein S, protein C, and antithrombin III (AT-III) should be measured to rule out any preexisting hypercoagulable state in patients with post-VZV CVST [8]. In this case, the diagnosis of post-primary VZV CVST was made on a clinical and radiological basis. In the ED, a CT scan revealed extensive dural sinus venous thrombosis.

Virus-associated vascular complications are frequently treated using intravenous acyclovir [7]. Furthermore, anticoagulation therapy with systemic heparin is usually the basis of treatment for acute CVST. However, poor outcomes in 9-13% of the patients being treated with anticoagulation therapy for CVST have been reported, as it may not dissolve the clot, and in a few occasions may deteriorate the patient's clinical condition. With anticoagulation alone, the rate of partial or complete recanalization ranged between 47-100%. Alternate treatments for CVST include fibrinolytic therapy, direct catheter thrombolysis, mechanical thrombolysis and thrombectomy, and surgical management. Anticoagulation therapy is usually continued following an acute CVST episode [8]. The patient was administered intravenous acyclovir and systemic heparin after the confirmation of diagnosis. After clinical improvement of the patient, heparin was then bridged to warfarin for chronic management of venous thrombosis.

## Conclusions

We conclude that the clinician should be aware of rare complications and their mechanisms so that a timely diagnosis can be made and prompt treatment can be initiated. Patients with primary VZV infection are prone to hypercoagulable states, owing to a decrease in natural anticoagulant levels. As a consequence, a fatal widespread thromboembolic phenomenon can occur. Prompt treatment with adequate hydration and anticoagulation can reduce mortality and improve the prognosis. Further studies need to be done to improve the understanding of CVST as a complication of primary VZV infection.  
